# Functional and Structural Features of Disease-Related Protein Variants

**DOI:** 10.3390/ijms20071530

**Published:** 2019-03-27

**Authors:** Castrense Savojardo, Giulia Babbi, Pier Luigi Martelli, Rita Casadio

**Affiliations:** 1 Biocomputing Group, Department of Pharmacy and Biotechnology, University of Bologna, 40126 Bologna, Italy; castrense.savojardo2@unibo.it (C.S.); giulia.babbi3@unibo.it (G.B.); rita.casadio@unibo.it (R.C.); 2 Institute of Biomembranes, Bioenergetics and Molecular Biotechnologies (IBIOM), Italian National Research Council (CNR), 70126 Bari, Italy

**Keywords:** genetic variations, disease-related protein variations, protein structure, polar solvent accessible surface, disease-related Pfam domains, disease-related reactome pathways

## Abstract

Modern sequencing technologies provide an unprecedented amount of data of single-nucleotide variations occurring in coding regions and leading to changes in the expressed protein sequences. A significant fraction of these single-residue variations is linked to disease onset and collected in public databases. In recent years, many scientific studies have been focusing on the dissection of salient features of disease-related variations from different perspectives. In this work, we complement previous analyses by updating a dataset of disease-related variations occurring in proteins with 3D structure. Within this dataset, we describe functional and structural features that can be of interest for characterizing disease-related variations, including major chemico-physical properties, the strength of association to disease of variation types, their effect on protein stability, their location on the protein structure, and their distribution in Pfam structural/functional protein models. Our results support previous findings obtained in different data sets and introduce Pfam models as possible fingerprints of patterns of disease related single-nucleotide variations.

## 1. Introduction

The elucidation of the association between individual phenotypes and personal genome is the major goal of modern genomic research. This research program is heavily supported by the fast improvement of sequencing technologies and it mostly focuses on the dissection of the genetic basis of diseases [1]. Although many phenotypic traits depend on the combination of multiple genetic and environmental factors, a number of relationships between single genes and diseases have been collected in databases [2,3]. Among the different types of variations, the most investigated are single-nucleotide mutations occurring in coding regions and leading to the substitution of a single residue in the expressed proteins. The challenge is then to assess the effects of single residue variations (SRVs) on protein structure and function [4]. Different studies searched for features that can help the discrimination of disease related variations in terms of type of mutation, localization on the protein structure, and functional characterization of the protein carrying the variation [5,6,7,8,9,10,11,12,13]. As a general trend, disease-related SRVs are often found in buried regions [9,10] or in surface patches likely to mediate interaction with other proteins [6,8], while a small number occurs in catalytic and metal binding sites [6,9]. Proteins carrying disease related mutations perform many different functions, and a relevant number of enzymes is involved [9].

Relation to protein stability has been investigated with different approaches and on different datasets. Since the thermodynamic effect of disease-related SRVs has been characterized in few cases [14,15,16], investigations often rely on computational methods for the prediction of protein stability perturbation [5,6,17,18]. Alternatively, the relation between stability perturbation and effect on disease has been investigated on the basis of the variation type (i.e., the pair of wild type and variant residues, independently of the position on the protein), by adopting standard substitution matrices [19], or matrices derived from experimental data on disease association or on stability perturbation induced by SRVs [20,21]. The general agreement is that disease-associated SRVs are often perturbing protein stability. Although significant, the correlation between the two effects is however only moderate [11,20,21,22].

In this work, we complement previous analyses by updating the dataset of disease-related SRVs occurring in proteins with known 3D structures. After mapping SRVs to protein structures, we describe all the functional and structural features that are of interest for characterizing disease-related SRVs. Our analysis highlights the chemico-physical features related to the most frequently occurring disease SRVs, and the molecular functions, pathways and structural/functional Pfam domains most affected by disease SVRs. Our results support previous findings in protein sets different from ours [8,9,20,21] and it adds to the notion that not all the disease related variation types are perturbing the protein stability [20,21]. Furthermore, mapping SVRs onto Pfam domains reveals that the models can be adopted as possible fingerprints of variation patterns, depending on the disease and associated protein function.

## 2. Results and Discussion

### 2.1. HVAR3D: A Dataset of Protein Variants with Structural and Functional Information

We extracted from Humsavar March 2018, (https://www.uniprot.org/docs/humsavar) a set of single residue variations (SRVs), comprising 30,221 disease-related and 39,894 neutral polymorphisms. Then, we mapped SRVs into available protein structures in the Protein Data Bank (PDB) (https://www.rcsb.org/), restricting our 3D set to some 2070 protein chains, of which 764 contains 8127 disease SVRs (Figure S1).

The number of protein structures mapping disease SRVs depends on the relative sequence to structure coverage (Figure S1), and only extended coverage (e.g., ≥70%) allows a correct analysis of their structural and functional features. When considering only proteins having at least one disease-related SRV and structure covering at least 70% of the corresponding UniProtKB sequence (with resolution lower than 3 Å), the PDB dataset reduces to 386 entries, including 1044 neutral and 5577 disease SRVs, in 392 protein chains (Table 1). We refer to this dataset as HVAR3D (Human Variants 3D). Our HVAR3D data set includes protein chains containing disease related (protein is labeled D) and disease-related and neutral (D/N) variations

### 2.2. Structural Characterization of HVAR3D

About 62% of the proteins in the data set have multimeric biological assemblies (Table 2). Some 43% of the multimers are homodimers. This allows mapping disease-related variations also at the interface region (Table 2). 

Following CATH structural classification (http://www.cathdb.info/), HVAR3D protein chains group into five structural classes (Table S1). Most of the protein chains group into the Alpha and Beta class, accounting for some 2207 disease related and 266 neutral variations. Disease-related variations similarly distribute in all the main structural classes (α, β and α + β).

As previously described [8,9], the contribution of missense mutations to human diseases is also due to specific interface residues which may affect complex stability when variated. For this reason, in our data set, we discriminate functional monomers from functional multimers, finding that about 9% of disease SRVs reside at interface (Table 2).

### 2.3. Functional Characterization of HVAR3D 

With the aim of highlighting some association among disease-related variations and variant structural and functional features, we first inspect which functional annotations are present in the data set. We extracted the enzyme commission (EC) numbers associated to the 392 protein chains (corresponding to the 386 entries) in HVAR3D dataset and counted, for each enzymatic class (i.e., first-digit EC number), the number of disease-related and neutral SRVs. Furthermore, we also count the frequency of appearance of an SRV in an annotated active or binding site (Table 3). We found that 248 out of 392 proteins (63%) are endowed with at least one EC number, accounting for 852 disease-related variations in 83 protein chains (D) and 3406 disease-related and 455 neutral variations in 165 protein chains (D/N). Among the different enzymatic classes, Oxidoreductases, Transferases and Hydrolases contain the highest numbers of protein chains and disease-related SRVs. Only seven protein chains are annotated with multiple ECs.

It appears that the number of disease-related variations falling into an active site or a ligand-binding site is only 1% of the total number of disease-related variations in the set. This figure is strictly dependent on the extent of annotation present in UniprotKB and it differs from previous studies where prediction methods were adopted [8,9].

To complement the above analysis, we further investigated the set of 144 protein chains lacking EC annotation by extracting, when available, the gene ontology (GO) molecular function (MF) terms. In doing this, we restricted our attention to the first-level GO molecular function terms (i.e., terms that are direct descendant of the GO molecular function ontology root (http://geneontology.org/page/download-ontology). The gene ontology lists 19 first-level molecular function terms: we found that 10 GO MFs are sufficient to annotate our 144 protein chains. One hundred and twenty protein chains can be annotated with the first level “binding (GO:0005488)” GO MF term: in 29 protein chains the term is the only annotation, whereas in 91 protein chains it couples with at least one of the remaining nine GO MFs (Table 4).

The 144 protein chains include 66 D protein chains mapping 319 disease related variations and 78 D/N protein chains mapping 1000 disease related and 589 neutral variations (Table 4), for a total sum of 1319 disease related and 589 neutral variations. Table 5 also lists disease-related and neutral SRVs, and the number of times SRVs of the two categories fall into a ligand-binding site. Similarly to what we find in the Enzyme set (Table 3), it appears that the number of variations falling in binding sites is a minor fraction of the total (1%).

Reactome pathways including protein chains of HVAR3D are listed in Table S3, according to the first-level biological pathways as extracted from the Reactome database (https://reactome.org/) (along with the number of associated disease-related and neutral variants). Nineteen out of 25 first level Reactome pathways are populated by proteins contained in the database (even in the case of the 97 protein chains with multiple Reactome pathways). Forty-four percent of the protein chains in HVAR3D are involved in metabolic pathways (collectively accounting for 168 chains and 2796 disease-related variations). The observation is consistent with the fact that 63% of the protein chains are enzymes. The second most populated Reactome first level pathway is the Immune system, accounting for 27 protein chains with 461 disease related variations (Table S2).

### 2.4. Structural and Functional Characterization of HVAR3D 

In HVARD3, 370 protein chains have 357 Pfam functional domains (https://pfam.xfam.org/), mapping some 4895 disease-related (87.7% of the total) and 798 neutral (76.4% of the total) variations.

Table 5 lists the most populated and the three less populated Pfam domains, the number of covered protein chains and the number of disease-related and neutral variations present in the domain. Fifty Pfam models contain 67.5% of disease SVRs in the data base (Figure S2).

Overall, HVAR3D is a database of fairly well categorized and annotated proteins offering an ample variety of structural and functional features to associate with disease-related variations. 

### 2.5. Chemico-Physical Characterization of Disease Related Variations in HVAR3D

We start our analysis following a long-standing discussion, aiming at understating whether possible features of disease-related variations can emerge from the change in chemico-physical properties of the lateral side chain [7,17,20,21]. In this regard, our database is a possible source of information, in which the position of the variation can immediately be evaluated in the context of the wild type protein structure. Our analysis investigates what HVAR3D contains in relation to the different types of variations. When necessary for statistical robustness, the number of neutral variations is increased by adopting a reference data set comprising 2667 neutral variations from 728 protein chains covering more than 70% of corresponding UniprotKB sequences (see Material and Methods).

**Figure 1:**
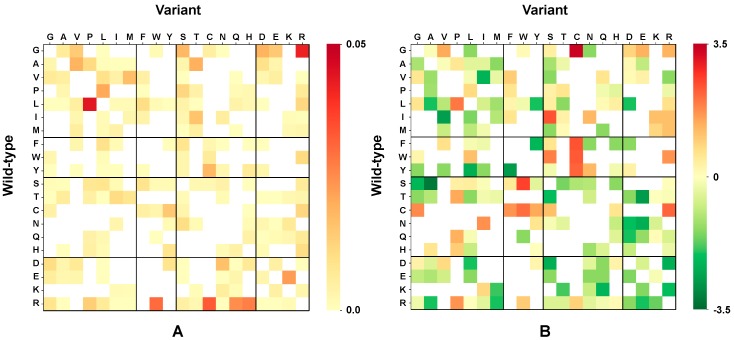
Disease-related variation types in HVAR3D. (**A**) The heatmap shows the frequency of occurrence of each variation type in the set of the disease-related variants. The color spans from yellow (low frequency) to red (high frequency); (**B**) The heatmap shows the association strength of each variation type to disease (Log-odd among frequencies of each variation type of the disease and neutral sets). Residues are grouped as follows: apolar (GAVPLIM), aromatic (FWY), polar (STCNQH), charged (DEKR), with residues in the same group listed at increasing order of size. The color code spans from green (variant types most frequent in the neutral set) to red (variant type most frequent in the disease related type). Computational methods are detailed in Materials and Methods.

In Figure 1A, the frequency of disease-related variation types emerges from the color code: the darker the red, the more frequent the disease-related variation type, the lighter the red the less frequent the disease-related variation type. In Figure 1B we show the measure of the strength of association (Log-odds ratio) of a given variation type to disease or neutral polymorphism (for details about the computational method, see Materials and Methods). 

The heatmap indicates that variations mostly associated to disease are G → C, followed by S→W, F→C, W→C, Y→C and I → S, whereas S→A, T→ E, Y→ F, F→Y are more strongly associated to neutral variations. Interestingly, the values of Figure 1B well correlates (0.71 of Pearson correlation) with our previous probability of having a variation associated to disease (P_d_ index) [20], derived from a much larger data set comprising some 5300 protein sequences and 9896 disease-related variations.

### 2.6. Correlation of Structural and Chemico-Physical Features of Disease SVRs in HVAR3D

#### 2.6.1. Protein Stability Perturbartion and Chemico-Physical Features of Disease SVRs

**Figure 2:**
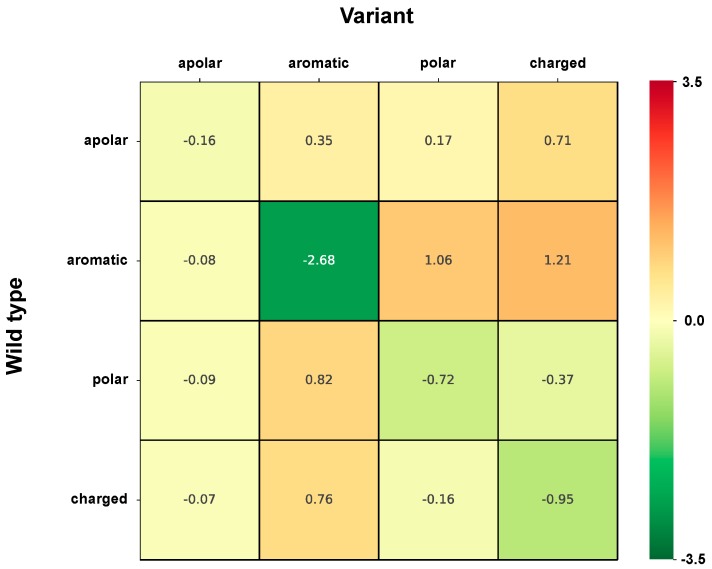
Heatmap shows the strength of association to disease (Log-odd) of variation types grouped according to chemico-physical properties of lateral side chains. Residues are grouped as following: apolar (GAVPLIM), aromatic (FWY), polar (STCNQH), charged (DEKR). The color code spans from green (variant types most frequent in the neutral set) to red (variant type most frequent in the disease related type). Estimated average standard error on reported values is 0.13.

For a better understanding of the effect of changing/conserving the physico-chemical properties of the variations, we group all the different possible variations in cells, labelled according to each of the major chemico-physical properties. Residues are grouped as following: apolar (GAVPLIM), aromatic (FWY), polar (STCNQH), charged (DEKR). Each one of the 16 cells indicates the strength of association to disease or to neutral polymorphisms of wild type lateral side chains of a given chemico-physical group turning into variations of the same or different chemico-physical groups. It appears that in HVAR3D, the variations mostly associated to disease are those turning an aromatic into a charged or polar residue, whereas when an aromatic residue turns into an aromatic residue, the association is towards neutral polymorphisms (Figure 2).

For sake of comparison with previous results, we investigated the relation among the type of variation and the extent of protein perturbation, as measured from the computed change in Gibbs free energy (ΔΔGs) upon single residue variation. To this aim, we adopted INPS-3D, a state-of-the-art prediction method previously described [20,23] and suited to compute the extent of protein stability change upon variation. INPS-3D computes ΔΔGs for all variations (disease and neutral) in our HVAR3D dataset complemented with the set of neutral variations in other 728 protein chains. Then, we divided the set of variations into two subsets: perturbing variations, having |ΔΔG| ≥ 1 kcal/mol and non-perturbing ones, with |ΔΔG|< 1 kcal/mol. 

**Figure 3:**
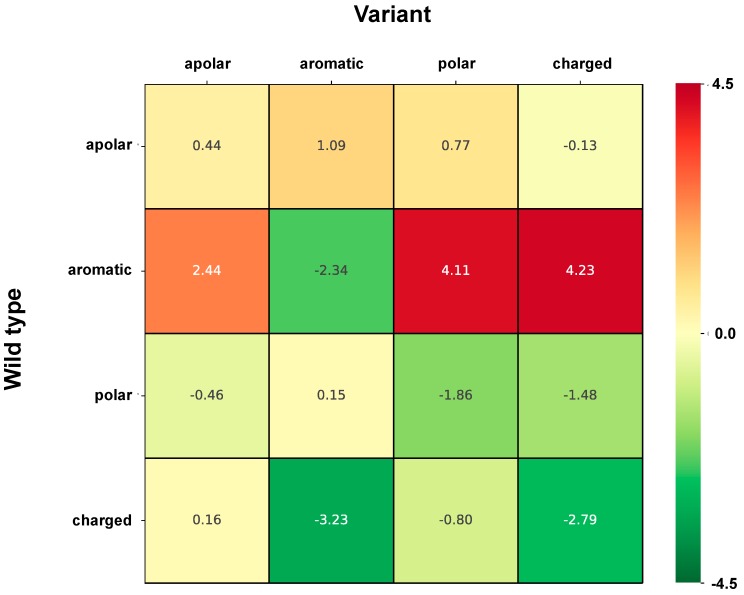
Heatmap shows the strength of association to perturbation (Log-odd) of variation types grouped according to chemico-physical properties of lateral side chains. Residues are grouped as following: apolar (GAVPLIM), aromatic (FWY), polar (STCNQH), charged (DEKR). The color code spans from green (variant types most frequent in the non-perturbing set) to red (variant type most frequent in the perturbing set). Estimated average standard error on reported values is 0.17.

Figure 3 shows the strength of association to perturbation of protein stability (Log-odd ratio, computed using the classification of variations into perturbing/non-perturbing) of the different variation types grouped according to their chemico-physical properties, similarly to Figure 2. It appears that the most perturbing variations correspond to a change from aromatic side chains into polar and charged ones and to a less extent also into apolar ones. Less perturbing variations are those where a charged residue turns into aromatic or charged lateral side chains. 

**Figure 4:**
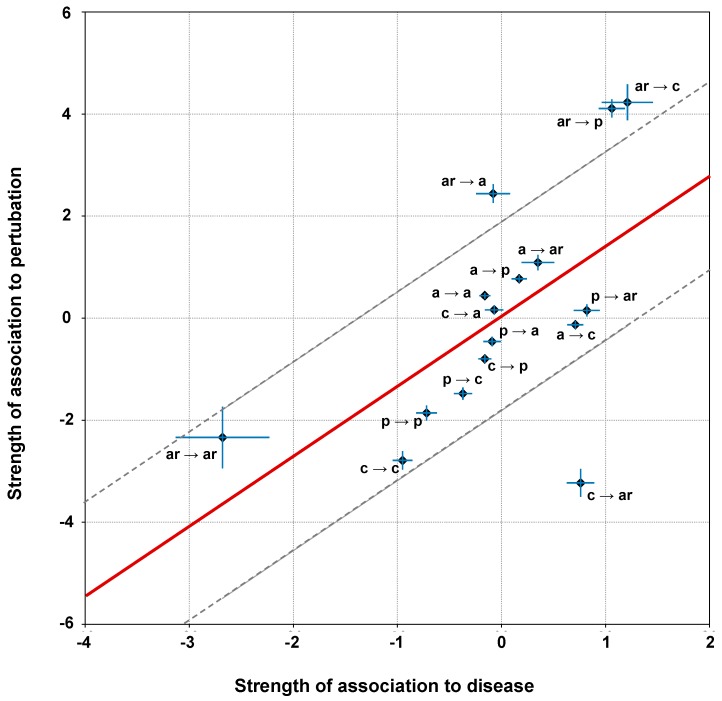
Correlation among strength of association of SRV types to diseases versus their strength of association to protein stability perturbation. Labels for variation types are as follows: p=polar, ar = aromatic, a = apolar and c = charged. Regression line is computed as described in Section 3.6. Error bars along both axes are computed as detailed in Section 3.5. Pearson’s correlation coefficient is equal 0.59 (*p*-value equals 0.01).

The regression plot of the association strengths depicted in Figure 2 and Figure 3, is presented in Figure 4. Each data point is reported with the associated standard errors alongside both axes. The regression line is shown in red, while grey dotted lines are drawn at an ordinate distance from the regression line equal to the root mean square of the residuals (RMSE = 1.89) (see Materials and Methods for details). 

Two main outliers can be identified: (i) variations from aromatic to polar residues, which are slightly associated to neutral polymorphisms (association strength is –0.08) but strongly associated to perturbation (association strength is 2.44); (ii) variations from charged to aromatic residues, which are moderately associated to disease (association strength is 0.76) but strongly not associated to perturbation (association strength is –3.23). Pearson’s correlation value is 0.59, suggesting, as previously reported [20], that the association of disease related variations to perturbing ones is moderate (Figure 4).

#### 2.6.2. Residue Solvent Exposure and Chemico-Physical Features of Disease SRVs 

**Figure 5:**
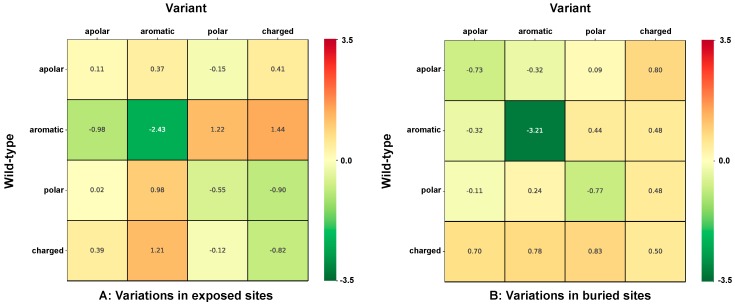
Heatmap shows the strength of association to disease (Log-odds ratio) of variation types in relation to solvent exposure. Variation types are grouped according to chemico-physical properties of lateral side chains. Residues are grouped as following: apolar (GAVPLIM), aromatic (FWY), polar (STCNQH), charged (DEKR). Panel (**A**) shows the association to disease of variations occurring in sites with relative solvent accessibility (RSA) ≥ 20%. Panel (**B**) shows the association to disease of variations occurring in sites with RSA < 20%. Estimated average standard errors on values in Panel (**A**) and (**B**) are 0.24 and 0.18, respectively.

In Figure 5, we analyze in more details whether the solvent exposure of the variated site influences the strength of the association between disease and variation type. We group variations as a function of their chemico-physical properties. Increasing red and green color intensities indicate that variation types are more associated to disease or neutral polymorphisms, respectively. The solvent exposed disease related variations are compared to the buried ones (Figure 5A,B, respectively). Major differences are: (i) apolar residues turning into apolar or aromatic ones are associated with disease when occurring in exposed sites and with neutral polymorphisms when they are located in buried sites; (ii) charged residues turning into polar or charged residues are associated with disease when occurring in buried and to neutral polymorphisms in exposed sites; (iii) similarly, polar residues turning into charged residues are associated with disease when occurring in buried and to neutral polymorphisms in exposed sites. 

These observations are consistent with the general view that on average, buried regions in proteins are routinely richer in apolar and aromatic residues, while polar solvent accessible surfaces include more polar and charged residues. From our data, it appears that the distribution density of disease-related variations differs depending on whether the variation types is or not exposed to the polar solvent. These results are consistent with previous observations [8,9,21].

#### 2.6.3. Pfam Protein Domains and Chemico-Physical Features of Disease SRVs

**Figure 6:**
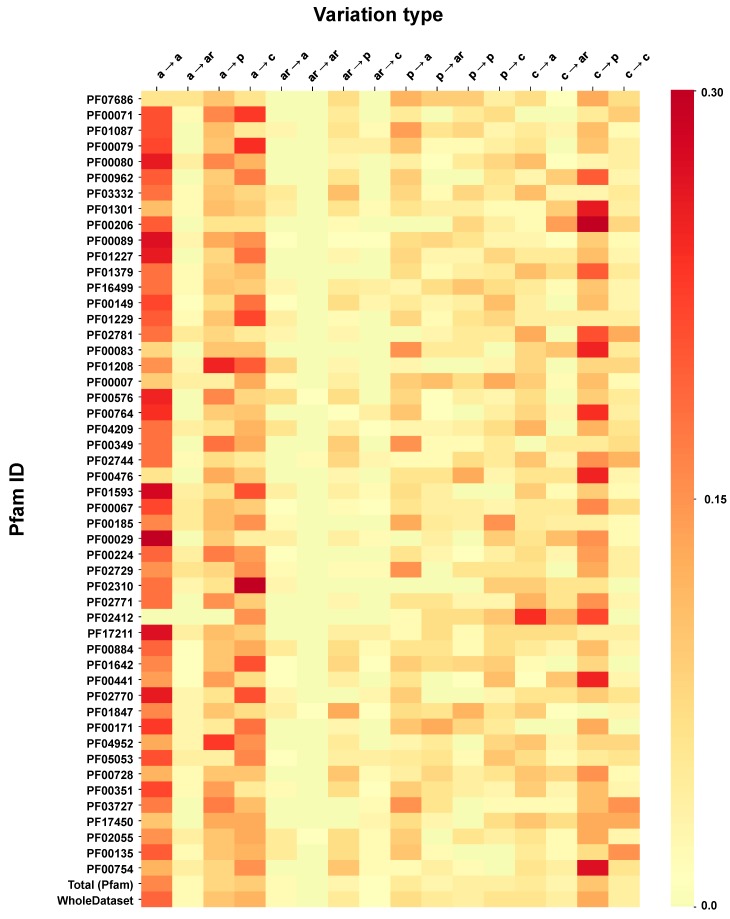
The heatmap shows the frequency of occurrence of each disease related variation type per Pfam domain. The color span from yellow (low frequency) to red (high frequency). Average and median Kullback–Leibler divergences between individual Pfam distributions and the background (WholeDataset) are 2.5 and 2.1 bits, respectively.

We analyze the distribution of SRVs chemico-physical types in Pfam domains and find out a useful representation. In Figure 6, the frequency of each disease SRV type is color coded with respect to Pfam domains which contain at least 30 variations. Fifty Pfam domains cover some 67.5% of the overall disease-related SRVs in HVAR3D and 87 protein chains (Figure S2). Disease SRV type frequency distribution (Figure 1A) is now associated to structural/functional Pfam domains. This makes it possible to retrieve per functional model where the most harmful MIM mutations are located at a structural level. As expected, disease SVR types are not homogenously distributed among the different Pfams. When compared to background distribution (last two rows of Figure 6, Total = contained in 357 Pfam domains, and whole data set), it appears that the most frequent disease variation type, apolar into apolar (top left quadrant of Figure 1A) is also spread among Pfams, with few exceptions. From the background distribution, PF02414, corresponding to Thrombospondin type 3 repeat, has disease-related variations when a charged residue turns into apolar, aromatic, and polar residues. The domain derives from PDB 3FBY, which represents in HVAR3D the Cartilage oligomeric matrix protein. In the corresponding P49747 (COMP_HUMAN) Uniprot file, 28 Aspartic acid residues (D) changing to apolar, aromatic and polar are associated to Pseudoachondroplasia and Multiple epiphyseal dysplasia 1, both Mendelian skeletal disorders. It also turns out that D residues in the native protein are ion Calcium stabilized. Another interesting example, is PF00476, DNA polymerase (EC: 2.7.7.7), which contains variations related to changes of lateral side chains from polar to polar and from charged to polar (3/3 and 3/4 quadrants from top left of Figure 1A, respectively). 

**Figure 7:**
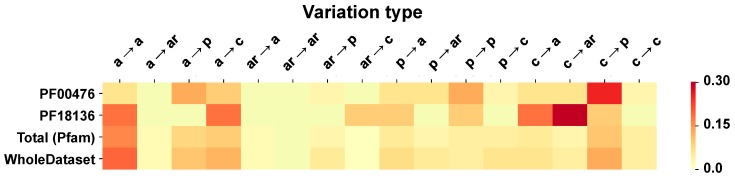
The heatmap shows the frequency of occurrence of each disease related variation type in each of the two Pfam domains of the DNA polymerase subunit gamma-1 (P54098 (DPOG1_HUMAN). The color span from yellow (low frequency) to red (high frequency). Kullback–Leibler divergence between distributions of the two Pfams domains is 2.5 bits.

In HVAR3D, this Pfam model represents protein 3IKM, the human mitochondrial DNA polymerase subunit gamma-1, (P54098, DPOG1_HUMAN), whose mutations are associated to seven different OMIM mitochondrial diseases (P54098). Interestingly, the protein (1239 residue long) is characterized by the presence of two domains, the C-terminal PF00476, containing the polymerase catalytic site, and the N-terminal PF18136, a DNA mitochondrial polymerase exonuclease domain, which acts as a proofreading exonuclease. This last domain is not included in Figure 6, for sake of brevity and it is shown in Figure 7, where the distribution of the disease variation types is compared. The protein contains 59 disease related variations, 30 in the first and 11 in the second Pfam domain. The remaining 18 variations are located in the interdomain region. Although the two Pfams share a common pattern of charged to apolar, aromatic and polar residue variations associated to disease, they differ for the apolar to apolar frequency that is relevant only in the DNA mitochondrial polymerase exonuclease (Figure 7). 

## 3. Materials and Methods

### 3.1. Variant Data Collection and Mapping to Protein Data Bank (PDB)

SRVs were collected from the release of March 2018 of the Humsavar. In order to perform structural and functional analyses, we firstly mapped SRVs from protein sequences on positions in corresponding PDB structures. For this task, we used the PDBSWS website (last updated on July 2018) [24] and SIFTS mapping [25]. PDB coverage was computed dividing the number of residues covered in the PDB by the length of the UniprotKB sequence deprived of signal or transit peptides, when present. 

In the HVAR3D dataset, we collected all disease-related and neutral variations occurring in protein chains covering more than 70% of the corresponding UniprotKB sequence and endowed with at least one disease-related variation (i.e., we retained only chains having both disease-related and neutral or only disease-related variations).

### 3.2. Retrieving Protein Annotations

Enzyme commission (EC) numbers and Gene Ontology Molecular Function (GO-MF) terms were retrieved for each protein using cross references directly available from the UniprotKB entry. In computing statistics for variations, all available EC numbers (at any level of annotation) were collapsed into their first-digit classification. 

For each GO-MF term annotated, we reconstructed the full path to the ontology root node using the GO-TermFinder Perl software package (https://metacpan.org/release/GO-TermFinder) [26] and using the latest version of GO obo ontology file available at the Gene Ontology Consortium website (http://geneontology.org/page/download-ontology). Complete DAGs for all GO-MF annotated on each protein were then merged together into a single functional annotation. Each protein chain (and corresponding variants therein) were then classified into one (or more) terms from the first-level GO-MF annotation (overall comprising 19 terms).

Positions of ACTIVE and BINDING sites for each protein chain were retrieved directly from features annotated on the UniprotKB entry.

Reactome pathway annotation was retrieved for each protein using the all-level pathway hierarchy mapping file (available at https://reactome.org/download-data). This file assigns, to each UniprotKB accession, the full Reactome annotation at all levels of the hierarchy. From these, we extracted the first-level pathways used to perform statistics. 

CATH classification was performed by downloading, from the CATH website (http://www.cathdb.info/), annotation file mapping CATH domains on UniprotKB/PDB entries.

Annotation of Pfam domains was derived from Pfam/UniprotKB mapping files available at the database website (ftp://ftp.ebi.ac.uk/pub/databases/Pfam/).

### 3.3. Computing Residue Surface Exposure and Protein-Protein Interfaces

We computed Absolute Solvent Accessibility (ASA) for each residue using the DSSP program [27]. Relative Solvent Accessibility (RSA) was computed by dividing the ASA value by the maximum theoretical accessible area as given in [28].

Protein complexes were analyzed in order to identify protein-protein interfaces. We defined protein-protein interaction sites as those residues that undergo a change ≥1Å^2^ in the accessible surface area upon formation of the complex.

### 3.4. Prediction of the Impact of SRVs on Protein Stability

The effect of different SRVs on protein stability has been computationally evaluated with INPS-MD [17], a method based on Support Vector Regression (SVR) for estimating the difference folding Gibbs free energy between the wild-type and the mutated forms of the proteins (ΔΔG). INPS-MD analyses two sets of descriptors extracted from protein sequence and structure, respectively. The former includes BLOSUM62 scores, hydrophobicity, Dayhoff mutability index and evolutionary information derived from multiple sequence alignments, while the latter consists of the residue relative solvent exposure and the difference between the native and the variant structure in terms of pairwise contact potential computed in a local structural environment (for further details, refer to [22,23]). When assessed on a dataset comprising 2648 variations whose ΔΔG has been experimentally determined, INPS-MD performs with a Pearson’s correlation index of 0.58 and a root mean error of 1.2 kcal/mol.

Each variation can be classified as perturbing or non-perturbing the protein stability according to the value of predicted ΔΔG. In particular, we define perturbing variations as those having |ΔΔG| ≥ 1, non-perturbing otherwise (i.e., |ΔΔG| < 1).

### 3.5. Computing the Association Strength of Each Variation Type to Disease and Pertubation

The association strength of each variation type to disease has been evaluated from the HVAR3D dataset. In order to strengthen our statistical analysis, HVAR3D was complemented using an additional dataset comprising 2667 neutral variations occurring on protein chains endowed with only neutral variations and covering more than 70% of the corresponding UniprotKB sequence (this set of neutral variations was not considered in all other analyses). Moreover, from disease-related variations in HVAR3D, we excluded those falling on ACTIVE or BINDING sites as well as those localized on known protein-protein interfaces.

After this procedure, we obtained a dataset of 8701 variations comprising 4990 and 3626 disease-related and neutral variations, respectively. We used this extended dataset to perform association analysis. 

Let V be the resulting set of variations comprising $N_{d}$ and $N_{n}$ disease-related and neutral variations, respectively. For each variation type X→Y from residue type X to Y, we can compute the frequencies of occurrence $f_{d}$ and $f_{n}$ in disease-related and neutral subsets, respectively, as follows:

(1) $$f_{d}=\frac{\#{\left(X\to Y\right)}_{d}}{N_{d}}$$

(2) $$f_{n}=\frac{\#{\left(X\to Y\right)}_{n}}{N_{n}}$$

where $\#{\left(X\to Y\right)}_{d}$ and $\#{\left(X\to Y\right)}_{n}$ are the number of occurrences of the variation type X→Y in the disease-related and neutral subsets, respectively.

The association strength $s_{X\to Y}^{d}$ (Log-odd ratio) of variation type X→Y to disease is then computed as:

(3) $$s_{X\to Y}^{d}=\log_{2}\frac{f_{d}}{f_{n}}$$

An estimate of the standard error on the log odd ratios in Equation (3) can be computed as follows:

(4) $$\mathrm{SE}\left(s_{X\to Y}^{d}\right)=\sqrt{\frac{1}{\#{\left(X\to Y\right)}_{d}}+\frac{1}{N_{d}}+\frac{1}{\#{\left(X\to Y\right)}_{n}}+\frac{1}{N_{n}}}$$

Analogously, associations of variation types to perturbation of protein stability has been evaluated on the same set V of variations as above (i.e., HVAR3D + complement of neutral variations). First, variations in V were classified into perturbing and non-perturbing using INPS-3D as explained in the Section 3.4. Then, we computed frequencies of occurrence $f_{p}$ and $f_{t}$ of variation type X→Y on the perturbing and non-perturbing subsets, respectively:

(5) $$f_{p}=\frac{\#{\left(X\to Y\right)}_{p}}{N_{p}}$$

(6) $$f_{t}=\frac{\#{\left(X\to Y\right)}_{t}}{N_{t}}$$

where $\#{\left(X\to Y\right)}_{p}$ and $\#{\left(X\to Y\right)}_{t}$ are the number of occurrences of the variation type X→Y in the perturbing and non-perturbing subsets, respectively, and $N_{p}$ and $N_{t}$ are the total number of perturbing and non-perturbing variations, respectively.

The association strength $s_{X\to Y}^{t}$ (Log-odd ratio) of variation type X→Y to perturbation is then computed as:

(7) $$s_{X\to Y}^{t}=\log_{2}\frac{f_{p}}{f_{t}}$$

Standard error is computed for $s_{X\to Y}^{t}$ as done for $s_{X\to Y}^{d}$ (Equation (4)).

### 3.6. Computing Linear Regression and Residuals

Regression lines were computed using the ordinary least square method as implemented in the Linear Regression model of the Python Sklearn package (https://scikit-learn.org). In regression plots, lines parallel to the regression line are drawn at an ordinate distance equal to the root mean square error (RMSE) of the residuals, defined as:

(8) $$RMSE=\sqrt{\frac{\sum {\left(y_{i}-y_{i}^{*}\right)}^{2}}{n-2}}$$

where $y_{i}$ is the i-th experimental variable, $y_{i}^{*}$ is the prediction of the variable as obtained by the linear regression model and n is the number of variables.

### 3.7. Kullback–Lieber Divergence Between Distributions

Differences between probability distributions are evaluated using the Kullback–Leibler divergence. This is defined between two discrete probability distributions *p* and *q* defined on the same probability space Χ as:

(9) $$D_{KL}(p||q)=-\underset{x\in Χ}{\sum }p\left(x\right)log_{2}\frac{q\left(x\right)}{p\left(x\right)}$$

## 4. Conclusions

In this paper, we introduce a database (HVAR3D) that collects 392 protein chains with a well solved 3D structure, covering at least 70% of the corresponding protein sequence. This allows mapping to the protein structures some 6621 variations, 5577 of which are disease-related.

The database allows a detailed statistical analysis of which type of variations are mostly associated with diseases, whether they are in the core or at the surface, and how they relate to protein structure and function. 

For the analysis, we group residues into four chemico-physical types and adopt a sixteen-item code to include all the possible single-residue variation types (Figure 1A). The strength of disease association for each variation type highlights that those mostly associated to disease in our dataset turn aromatic into charged or polar residues (Figure 2), confirming previous observation in different data sets [7,9,20,21]. The correlation among the strength of association to disease and the strength of association to perturbation is significant (Pearson’s correlation value is 0.59), however moderate [20,21,29]. 

When variation types are sorted out by being on the protein surface or buried, we find that apolar residues turning into apolar or aromatic residues are more associated with disease than when they occur in exposed sites; in turn, they are more associated with neutral polymorphisms when they occur in buried sites [8]. 

We map disease SRV types to Pfam domains and find that their distribution varies across different structural/functional models. This suggests that the newly introduced mapping can be adopted to define each Pfam a specific a priori distribution patterns of disease related SRV types. 

## Figures and Tables

**Table 1:** Summary statistics of the HVAR3D dataset.

#PDB Entries	#SRVs	# SRVs Buried ^(b)^	# SRVs Exposed ^(c)^
386 (D+D/N) ^(a)^	6621	3998	2623
241 (D/N) ^(a)^	5450	3325	2125
145 (D) ^(a)^	1171	673	498

^(a)^ D = protein chains with only disease related variations, D/N = protein chains with both Disease related and Neutral variations; ^(b)^ Occurring at positions having relative solvent accessibility, RSA < 20%; ^(c)^ Occurring at position having RSA ≥ 20%. SRVs: Single Residue Variations.

**Table 2:** Biological assemblies in HVAR3D.

Biological Assembly	#PDB Entries	#D-SRVs ^(a)^	#N-SRV ^(b)^	#D-SRVs Interface ^(c)^	#N-SRVs Interface ^(d)^
Monomers	146	2011	340	-	-
Multimers	240	3566	704	501	109

^(a)^ D-SRVs = disease-related SRVs; ^(b)^ N-SRV = neutral SRVs; ^(c)^ Disease-related SRVs at the interface; ^(d)^ Neutral SRVs at the interface. For details on how to define the interface region see the Materials and Methods section. The total number of residues in monomers is 54107; the total number of residues in multimers is 72012, 10678 of which are protein-protein interaction sites.

**Table 3:** Enzymes in HVAR3D.

EC^(a)^	#Protein Chains	#D-SRVs ^(b)^	#N-SRVs ^(c)^	#D-SRVs on:		#N-SRVs on:	
				ACTIVE SITE	BINDING SITE	ACTIVE SITE	BINDING SITE
Oxidoreductases	64	1045	107	2	20	0	0
Transferases	71	1058	122	5	20	0	0
Hydrolases	69	1342	143	7	10	0	1
Lyases	19	260	36	0	3	0	0
Isomerases	7	236	14	0	7	0	0
Ligases	11	138	15	0	7	0	2
Multiple ECs	7	179	18	0	0	0	0
**Total**	**248**	**4258**	**455**	**14**	**67**	**0**	**3**

^(a)^ EC = enzyme commission; ^(b)^ D-SRVs = disease-related SRVs; ^(c)^ N-SRV = neutral SRVs. Residue annotation (Active site, Binding site) is taken from the corresponding protein file in UniprotKB.

**Table 4:** HVAR3D: Functional characterization of 144 protein chains lacking enzyme commission (EC) numbers.

GO MF ^(a)^	#Protein Chains	#D-SRVs	#N-SRVs	#D-SRVs on Binding Sites	#N-SRVs on Binding Sites
Antioxidant activity	1 (1) ^(b)^	3	55	0	1
Catalytic activity	22 (20)	291	52	0	0
Molecular carrier activity	2 (2)	11	279	0	11
Molecular function regulator	25 (22)	266	50	2	0
Molecular transducer activity	4 (3)	10	13	0	0
Structural molecule activity	11 (4)	167	19	0	0
Transcription regulator activity	4 (3)	13	2	0	0
Translation regulator activity	1 (1)	2	0	0	0
Transporter activity	10 (9)	134	27	3	0
Binding ^(c)^	29	297	29	0	0
Multiple terms ^(d)^	31 (26)	115	62	0	0
Non annotated	4	10	1	0	0
**Total**	**144 (120)**	**1319**	**589**	**5**	**12**

^(a)^ First Level gene ontology (GO) molecular function (MF); ^(b)^ (x) = number of protein chains that have also the first level term “binding (GO:0005488)”; ^(c)^ Statistics for the 29 protein chains having only the “binding (GO:0005488)”; ^(d)^ Statistics for protein chains endowed with multiple GO MF annotations.

**Table 5:** Pfam domains in HVAR3D. The highest and the lowest represented Pfam domains in terms of number of variations are reported in the first and last three rows, respectively.

Pfam ID	Pfam Name	#Protein Chains	#D-SRVs	#N-SRVs
PF00351	Biopterin-dependent aromatic amino acid hydroxylase	3	213	5
PF00884	Sulfatase	4	159	4
PF16499	Alpha galactosidase A	2	143	15
PF00023	Ankyrin repeat	1	1	2
PF00050	Kazal-type serine protease inhibitor domain	1	1	2
PF00070	Pyridine nucleotide-disulphide oxidoreductase	1	1	0

^(a)^ Number of disease-related variants perturbing protein stability; ^(b)^ Number of neutral variants perturbing protein stability.

## Supplementary Materials

Supplementary materials can be found at https://www.mdpi.com/1422-0067/20/7/1530/s1.

## Abbreviations

SRV	Single Residue Variant
GO	Gene Ontology

## References

[1] Chakravorty S., Hegde M. (2017). Gene and variant annotation for mendelian disorders in the era of advanced sequencing technologies. Annu. Rev. Genom. Hum. Genet..

[2] Amberger J.S., Hamosh A. (2017). Searching online mendelian inheritance in man (OMIM): A knowledgebase of human genes and genetic phenotypes. Curr. Protoc. Bioinform..

[3] Babbi G., Martelli P.L., Profiti G., Bovo S., Savojardo C., Casadio R. (2017). eDGAR: A database of Disease-Gene Associations with annotated Relationships among genes. BMC Genom..

[4] Kroncke B.M., Vanoye C.G., Meiler J., George A.L., Sanders C.R. (2015). Personalized biochemistry and biophysics. Biochemistry.

[5] Wang Z., Moult J. (2001). SNPs, protein structure, and disease. Hum. Mutat..

[6] Steward R.E., MacArthur M.W., Laskowski R.A., Thornton J.M. (2003). Molecular basis of inherited diseases: A structural perspective. Trends Genet..

[7] Petukh M., Kucukkal T.G., Alexov E. (2015). On human disease-causing amino acid variants: Statistical study of sequence and structural patterns. Hum. Mutat..

[8] David A., Sternberg M.J. (2015). The contribution of missense mutations in core and rim residues of protein-protein interfaces to human disease. J. Mol. Biol..

[9] Gao M., Zhou H., Skolnick J. (2015). Insights into disease-associated mutations in the human proteome through protein structural analysis. Structure.

[10] Martelli P.L., Fariselli P., Savojardo C., Babbi G., Aggazio F., Casadio R. (2016). Large scale analysis of protein stability in OMIM disease related human protein variants. BMC Genom..

[11] Schaafsma G.C.P., Vihinen M. (2017). Large differences in proportions of harmful and benign amino acid substitutions between proteins and diseases. Hum. Mutat..

[12] Schaafsma G.C.P., Vihinen M. (2018). Representativeness of variation benchmark datasets. BMC Bioinform..

[13] Medina-Carmona E., Fuchs J.E., Gavira J.A., Mesa-Torres N., Neira J.L., Salido E., Palomino-Morales R., Burgos M., Timson D.J., Pey A.L. (2017). Enhanced vulnerability of human proteins towards disease-associated inactivation through divergent evolution. Hum. Mol. Genet..

[14] Khoo K.H., Mayer S., Fersht A.R. (2009). Effects of stability on the biological function of p53. J. Biol. Chem..

[15] Khoo K.H., Andreeva A., Fersht A.R. (2009). Adaptive evolution of p53 thermodynamic stability. J. Mol. Biol..

[16] Pey A.L., Megarity C.F., Timson D.J. (2019). NAD(P)H quinone oxidoreductase (NQO1): An enzyme which needs just enough mobility, in just the right places. Biosci. Rep..

[17] Yue P., Li Z., Moult J. (2005). Loss of protein structure stability as a major causative factor in monogenic disease. J. Mol. Biol..

[18] Laimer J., Hofer H., Fritz M., Wegenkittl S., Lackner P. (2015). MAESTRO—Multi agent stability prediction upon point mutations. BMC Bioinform..

[19] Ferrer-Costa C., Orozco M., de la Cruz X. (2002). Characterization of disease-associated single amino acid polymorphisms in terms of sequence and structure properties. J. Mol. Biol..

[20] Casadio R., Vassura M., Tiwari S., Fariselli P., Martelli P.L. (2011). Correlating disease-related mutations to their effect on protein stability: A large-scale analysis of the human proteome. Hum. Mutat..

[21] Peng Y., Alexov E. (2016). Investigating the linkage between disease-causing amino acid variants and their effect on protein stability and binding. Proteins.

[22] Fariselli P., Martelli P.L., Savojardo C., Casadio R. (2015). INPS: Predicting the impact of non-synonymous variations on protein stability from sequence. Bioinformatics.

[23] Savojardo C., Fariselli P., Martelli P.L., Casadio R. (2016). INPS-MD: A web server to predict stability of protein variants from sequence and structure. Bioinformatics.

[24] Martin A.C.R. (2005). Mapping PDB chains to UniProtKB entries. Bioinformatics.

[25] Velankar S., Dana J.M., Jacobsen J., van Ginkel G., Gane P.J., Luo J., Oldfield T.J., O’Donovan C., Martin M.J., Kleywegt G.J. (2013). SIFTS: Structure Integration with Function, Taxonomy and Sequences resource. Nucleic Acids Res..

[26] Boyle E.I., Weng S., Gollub J., Jin H., Botstein D., Cherry J.M., Sherlock G. (2004). GO::TermFinder—Open source software for accessing gene ontology information and finding significantly enriched gene ontology terms associated with a list of genes. Bioinformatics.

[27] Kabsch W., Sander C. (1983). Dictionary of protein secondary structure: Pattern recognition of hydrogen-bonded and geometrical features. Biopolymers.

[28] Rost B., Sander C. (1994). Conservation and prediction of solvent accessibility in protein families. Proteins.

[29] Niroula A., Vihinen M. (2016). Variation interpretation predictors: Principles, types, performance, and choice. Hum. Mutat..

