# Supplementary material for: Functional and Structural Features of Disease-Related Protein Variants
Source: Int J Mol Sci. 2019 Mar 27;20(7):1530. doi: 10.3390/ijms20071530 (PMC6479756; doi:10.3390/ijms20071530)
Supplement: Supplementary file 1 [file ijms-20-01530-s001.pdf]

Table S1. HVAR3D: Protein Chains and their variations as a function of the CATH structural classes.

CATH Class	#Protein Chains	#D-SRVs	#N-SRVs
Mainly alpha	42	606	428
Mainly beta	42	637	79
Alpha and beta	167	2207	266
Few secondary structure	1	1	1
Multi-class	130	1955	250
Non annotated	10	131	20
Total	392	5577	1044

CATH Protein structural clsssification (http://www.cathdb.info/); # Disease and Neutral single residue variation.

Table S2. HVAR3D: the Reactome pathways.

Reactome Pathway	#Protein Chains	#D-SRVs	#N-SRVs
Cell cycle	2	3	0
Cell-cell communication	1	12	2
Cellular responses to external stimuli	1	1	1
Developmental biology	1	1	7
DNA repair	7	20	23
Extracellular matrix organization	2	66	3
Gene expression	4	94	21
Hemostasis	2	7	31
Immune system	27	461	55
Metabolism	134	2349	220
Metabolism of proteins	28	425	46
Metabolism of RNA	6	22	1
Muscle contraction	3	22	8
Neural system	6	51	13
Organelle biogenesis and maintenance	4	31	4
Protein localization	3	143	8
Signal transduction	14	73	15
Transport of small molecules	10	132	37
Vesicle-mediated transport	3	38	14
Multiple REACTOME codes	97	1241	494
Non annotated	37	385	41
Total	392	5577	1044

Reactome Pathway database: https://reactome.org/

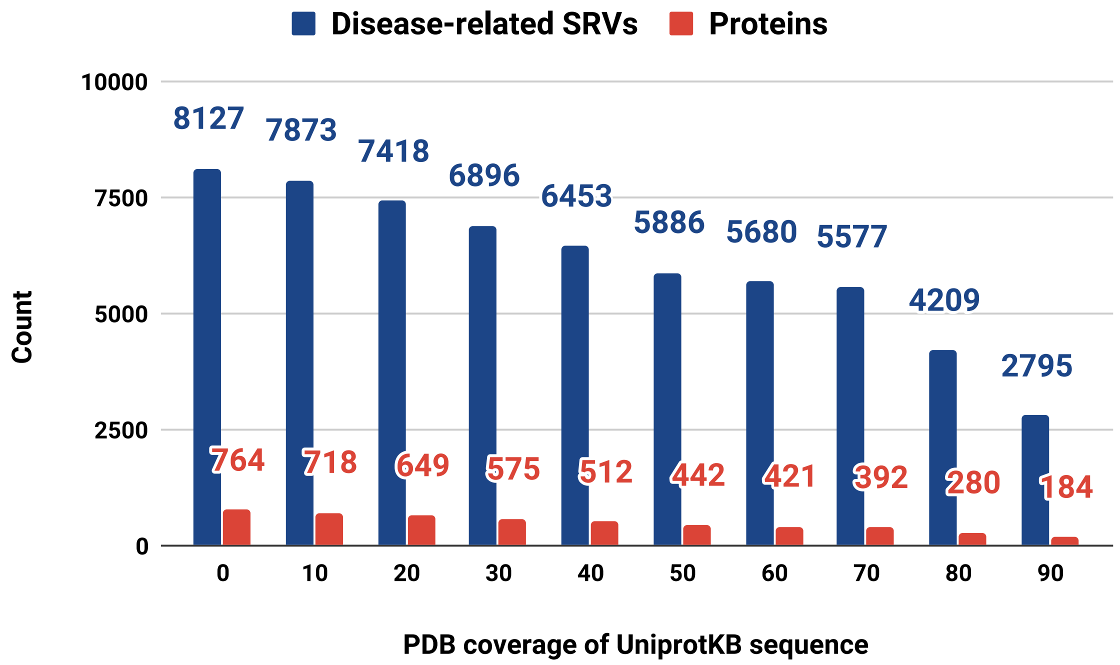

Figure S1. Disease related single residue variations as a function of the PDB structure to UnprotKB corresponding sequence. Blue numbers are SVRs and red ones are protein chains.

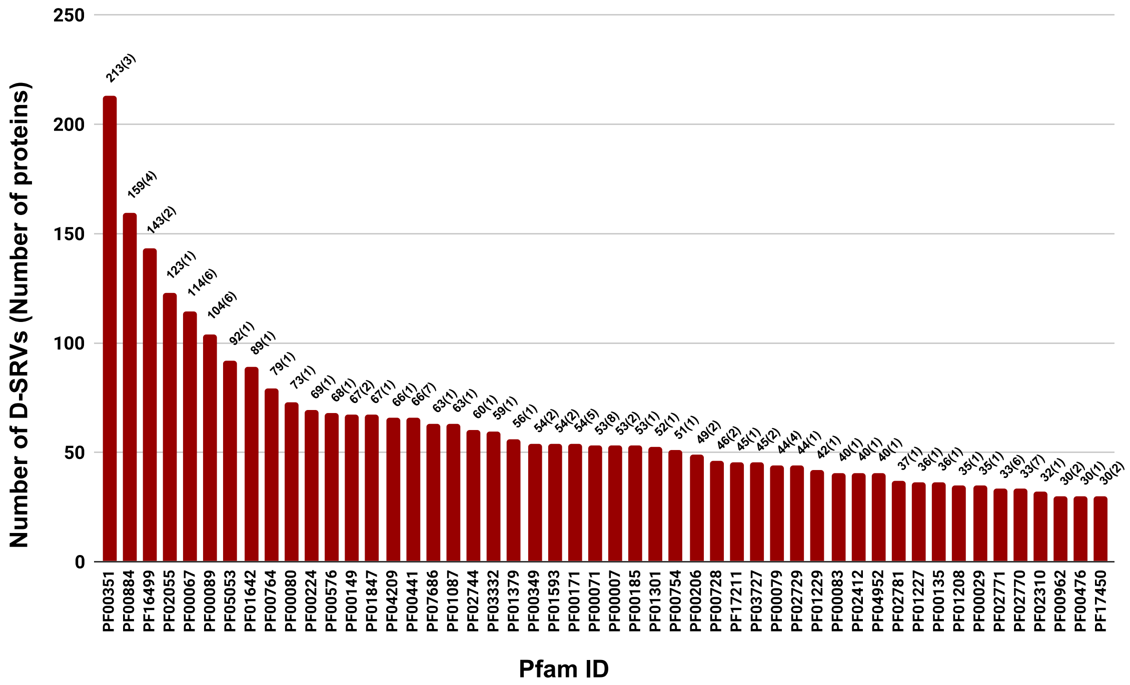

Figure S2. Bar plot distribution of disease SVRs in protein chains (number) per Pfam domain. Only 50 Pfam domains covering at least 30 SRVs, of the total 357 domains in HVAR3D, are shown.
